# Brain Activation and Psychomotor Speed in Middle-Aged Patients with Type 1 Diabetes: Relationships with Hyperglycemia and Brain Small Vessel Disease

**DOI:** 10.1155/2016/9571464

**Published:** 2016-02-21

**Authors:** Misun Hwang, Dana L. Tudorascu, Karen Nunley, Helmet Karim, Howard J. Aizenstein, Trevor J. Orchard, Caterina Rosano

**Affiliations:** ^1^Department of Radiology, University of Pittsburgh, 3600 Forbes Avenue, Plaza Level, Pittsburgh, PA 15213, USA; ^2^Department of Internal Medicine, Department of Psychiatry, and Department of Biostatistics, University of Pittsburgh, 200 Meyran Avenue, Suite 326, Pittsburgh, PA 15213, USA; ^3^Department of Epidemiology, University of Pittsburgh, 130 N. Bellefield Avenue, Suite 443, Pittsburgh, PA 15213, USA; ^4^Department of Bioengineering, University of Pittsburgh, 253 Sterling Plaza, Pittsburgh, PA 15213, USA; ^5^Department of Psychiatry, University of Pittsburgh, 3811 O'Hara Street, Pittsburgh, PA 15213, USA; ^6^Department of Epidemiology, University of Pittsburgh, 3512 Fifth Avenue, Pittsburgh, PA 15213, USA; ^7^Department of Epidemiology, University of Pittsburgh, 130 N. Bellefield Avenue, Suite 467, Pittsburgh, PA 15213, USA

## Abstract

Slower psychomotor speed is very common in patients with type 1 diabetes mellitus (T1D), but the underlying mechanisms are not clear. We propose that hyperglycemia is associated with slower psychomotor speed via disruption of brain activation. Eighty-five adults (48% women, mean age: 49.0 years, mean duration: 40.8) with childhood onset T1D were recruited for this cross-sectional study. Median response time in seconds (longer = worse performance) and brain activation were measured while performing a psychomotor speed task. Exposure to hyperglycemia, measured as glycosylated hemoglobin A1c, was associated with longer response time and with higher activation in the inferior frontal gyrus and primary sensorimotor and dorsal cingulate cortex. Higher activation in inferior frontal gyrus, primary sensorimotor cortex, thalamus, and cuneus was related to longer response times; in contrast, higher activation in the superior parietal lobe was associated with shorter response times. Associations were independent of small vessel disease in the brain or other organs. In this group of middle-aged adults with T1D, the pathway linking chronic hyperglycemia with slower processing speed appears to include increased brain activation, but not small vessel disease. Activation in the superior parietal lobe may compensate for dysregulation in brain activation in the presence of hyperglycemia.

## 1. Introduction

Compared to similarly aged nondiabetic adults, persons with type 1 diabetes (T1D) are more likely to exhibit cognitive deficits, especially localized within the psychomotor and information processing domains [1–5]. Slower psychomotor speed is associated with several negative health outcomes, in persons both with [6–8] and without diabetes [9, 10]. Deficits in psychomotor speed appear to increase as persons with T1D age and/or live longer with diabetes [11–13]. With the rising number of persons with T1D now surviving into older age [14], it is increasingly important to characterize the predictors of slower psychomotor speed to design interventions that reduce its negative impact on health outcomes.

The mechanisms underlying slower psychomotor speed in T1D are not entirely clear. Chronic hyperglycemia has been implicated in cognitive complications in several studies of T1D, including ours [15–18]. It has been suggested that chronic hyperglycemia may compromise cognitive function because of its detrimental effects on the microvasculature [18–20]. Indeed, small vessel diseases, including brain white matter hyperintensities and retinopathy, have been associated with slower psychomotor speed in persons with T1D [1, 21–24]. We have recently shown that white matter hyperintensities partially explain the slower psychomotor speed observed in middle-aged adults with T1D as compared to similarly aged non-T1D adults [25].

One hypothesized pathway linking chronic hyperglycemia, brain small vessel disease, and slower psychomotor speed could be via disrupted neuronal activation. Thickened cerebral cortical capillary basement membranes in diabetics and decreased capillary density are two hallmark signs of small vessel disease [26] which can potentially reduce oxygen delivery and vasoreactivity [27] and therefore alter neuronal activation. Moreover, positive relationships between imaging signs of small vessel disease and higher brain activation have been recently reported in neuroimaging studies of older adults [28–30] and in one small study of selected patients with T1D, using retinal imaging [20].

We propose that hyperglycemia affects psychomotor speed via disruption of brain activation and that brain small vessel disease mediates these associations. To test this hypothesis, we measured patterns of brain activation while performing a task of psychomotor speed, concurrently with historic measures of exposure to hyperglycemia and small vessel disease in the brain and other target organs.

## 2. Methods

### 2.1. Participants

In 2010–2013, individuals from the Pittsburgh Epidemiology of Diabetes Complications Study (EDC) were invited to a neurovascular ancillary imaging study. All participants had childhood-onset (<17 years of age) T1D and were diagnosed or seen within one year of diagnosis (1950–1980) at Children's Hospital of Pittsburgh. Following the first clinical assessment (1986–1988), when average participant age and diabetes duration were 28 and 19 years, respectively, biennial examinations were conducted for 10 years, with further examinations at 18 and 25 years [31]. Among the 263 locally resident individuals who participated in the 24-year EDC followup and were invited for this neurovascular imaging study, 149 accepted the invitation, 112 of whom did not have contraindication for brain MRI. Brain MRIs were obtained on *n* = 106 participants, and 89 of these had both functional brain MRI and performance measures while performing the task in the scanner. Of these, 85 also had complete cognitive data obtained outside the scanner and are included in this analysis.

All study procedures were approved by the Institutional Review Board at University of Pittsburgh Medical Center.

### 2.2. Measures of Interest

Prevalence of diabetes-related complications and other health and lifestyle factors were assessed periodically from 1986–1988 to 2004–2006 via questionnaire, physical exam, and medical records [31].

#### 2.2.1. Hyperglycemia

Glycosylated hemoglobin A1c (HbA1c) was measured at time of brain MRI and also at repeated intervals since study entry, using automated high-performance liquid chromatography as previously described [32]. A measure assessing degree and duration of hyperglycemia is computed as “HbA1c months” (for details, see [32]). HbA1c is a well-established marker of mean glucose concentrations during the prior 2-3 months [33].

#### 2.2.2. Brain MRI Protocol

Brain MRI scanning was conducted at the Magnetic Resonance Research Center at the University of Pittsburgh using a 3-Tesla Siemens Trio TIM scanner, with a 12-channel head coil. Structural scans were acquired prior to functional scans. Details on the protocol of acquisition are described in greater detail elsewhere [34]. A fully automated method for quantifying and localizing white matter hyperintensities on MR images was used. The algorithm uses a fully automated fuzzy connectedness algorithm. Seeds are generated from the histogram of the FLAIR image and thus user input is not needed compared to other algorithms. Each seed has its own parameters (providing more specific segmentation) which are calculated automatically (fuzzy adjacency and affinity) [35]. Magnetization-prepared rapid gradient echo (MPRAGE) T1-weighted images were acquired in the axial plane: TR = 2300 ms; TE = 3.43 ms; TI = 900 ms: flip angle = 9°; slice thickness = 1 mm; FOV = 256 mm × 224 mm; voxel size = 1 mm × 1 mm; matrix size 256 × 240; number of slices = 176. T2-weighted and FLAIR images were acquired as previously described [25].

#### 2.2.3. Functional MRI Paradigm

In this study, the computerized version of the Digit Symbol Substitution Tests (DSST) [36, 37] was adapted for the scanning procedure to test psychomotor speed, following a protocol described previously [38, 39]. The paradigm tests motor speed, response selection [40], shifting of attention [41], and perceptual speed [42, 43]. Psychomotor speed is used to actively maintain and manipulate information over a brief period of time and to allocate attentional resources among competing subtasks [44, 45]. Frontal and parietal lobes, thalamus, precuneus, and cingulate cortex are expected to be involved in performing these tasks [38, 39, 46, 47]. For this protocol, the subject sees one number-symbol matching pair (cue) on a computer screen. After the cue disappears, an answer key (probe) appears containing a grid of four number-symbol matching pairs. The subject is instructed to push the right index finger button if the probe contains a number-symbol that matches the cue or to push the left index finger button if the probe does not contain a number-symbol that matches the cue. Instructions are to respond “as fast as you possibly can.” Participants were instructed on the task outside the magnet for as long as needed to familiarize them with the task (usually 5–10 min). The DSST was presented using a block design with 8 trials/block, in which a block of the experimental DSST condition is alternated with control condition for a total of 10 blocks: 5 DSST blocks alternating with 5 control blocks for a total of 9 min and 20 s. Each block lasted for 56 s including 8 s of instructions at the start of each block to remind the subjects of the task. The 8 s of instruction and the first trial of each block were excluded from the analyses. The different task conditions (matching versus nonmatching) were randomized 1 : 1 across the block. The nonmatching condition was designed as a control condition to account for the non-task-specific brain activation, frontal eye field and visual cortex for eye movements and motor cortex for the index fingers movement, which are likely to be elicited during DSST performance in addition to the task-specific activation of the executive control function network. Responses occurring after the probe disappeared (after 3.6 s) were termed as “no response” and were omitted from the calculation of accuracy and response time. Median response time during the task was computed as the interval of time between appearance of the probe and the subject's response. Accuracy was recorded as number of correct responses divided by total number of responses [38, 39].

#### 2.2.4. Small Vessel Disease in the Brain

Total white matter hyperintensities (WMHs) volume was calculated using a previously published automated method [35, 48] and normalized for brain volume. Burden of WMH was also measured using Fazekas ratings as previously described [25].

#### 2.2.5. Small Vessel Disease in Other Target Organs

*Eye Disease*. Proliferative retinopathy was assessed by three-standard field stereo fundus photography at each EDC examination by the Madison Reading Center in Wisconsin and graded according to the ETDRS Classifications. A history of laser therapy for proliferative retinopathy is used as a proxy in the absence of photos.

*Neuropathy*.* Distal symmetric polyneuropathy* is determined by medical history and clinical examination using the DCCT protocol (i.e., the presence of two or more of the following: symptoms (consistent with DSP), reduced deep tendon reflexes, and signs of sensory loss) and it is further “confirmed” by determining age specific abnormal vibratory thresholds (Vibratron II) of the great toe.* Cardiac autonomic neuropathy* is assessed by heart variation during deep breathing and heart rate and BP response to standing.

*Renal Disease*.* Overt nephropathy* (ON), defined as the presence of* renal failure *(serum creatinine >5 mg/dL and/or ESRD, i.e., dialysis and/or renal transplant) or an albumin excretion rate >200 *μ*g/min in at least 2 of 3 timed urine samples or (in the absence of any urine collections) serum creatinine >2 mg/dL and* microalbuminuria*, defined as an albumin excretion rate between 20 and 200 *μ*g/min in at least 2 or 3 urine samples. If the adequacy of timed urine samples is questionable in terms of creatinine excretion, a previously validated urinary albumin to creatinine ratio (mg/gm) was used (i.e., >0.30 ON; 0.03–0.29 microalbuminuria).

#### 2.2.6. Cognitive Function

Participants were evaluated using a comprehensive battery of tests [17]: estimated verbal intelligence (North American Adult Reading Test [NAART]); psychomotor efficiency (Digit Symbol Substitution Test [DSST] and Grooved Pegboard); executive function (Trail Making Test, Part B [TMTB], Verbal Fluency [VF: animal naming and F-A-S], Stroop Color-Word, and Letter/Number Sequencing [LN Sequence]); learning and working memory (Rey Auditory Verbal Learning Tests [RVLT], Four Word Short-Term Memory [4WSTM] and Rey Osterrieth Complex Figure Delayed task [ROCF-delay]); and visuoconstruction skills (Rey Osterrieth Complex Figure Copy task [ROCF-copy]). Details on each of these tests can be found elsewhere (see Strauss et al. [49]). Individual raw test scores that were ≥1.5 SD worse than demographically appropriate published norms [49–51] were classified as “impaired” and impairment indicator variables were created for each test [17].

### 2.3. Statistical Analysis

Associations between sample characteristics and median response time while performing the task in the scanner were tested using Spearman or Pearson correlation coefficients depending on whether the distribution of the variables of interest was normal. The association of HbA1c months with median reaction time was tested in multivariable linear regression models after adjustment for markers of small vessel disease (brain WMHs, neuropathy, nephropathy, and retinopathy, and cardiac autonomic neuropathy). Since the markers of small vessel disease are highly correlated with each other, they entered the model separately. Small vessel disease in the brain was considered a potential mediator of the association between HbA1c and functional activation. Small vessel complications outside the brain can represent peripheral biomarkers of underlying brain damage; and were therefore considered confounders of the association between hyperglycemia and functional activation. The change in the regression coefficient of HbA1c months predicting median reaction time after adjusting for the above variables was measured; a change of >10% was considered as an indication that small vessel complications substantially attenuated the main association [52]. Models were further adjusted for the sample characteristics associated with median reaction time in univariate models.

Functional imaging data were analyzed with Statistical Parametric Mapping 5 (SPM5) using general linear models [53]. For each subject, all Epipolar-Plane Image (EPI) volumes were realigned to the first volume of the time series. A standard SPM high pass filter (1/128 hz) was applied. Smoothing was done at FWHM = 8 mm; physiological noise correction was performed. Each condition was modeled using a delayed boxcar function convolved with the SPM hemodynamic response function. Movement parameters derived from realignment were added as covariates of no interest to correct for confounding effects induced by head movement. Contrasts of interest (DSST > control and control > DSST) were first estimated for each subject individually averaging activation across runs. The resulting statistical maps with the contrast of interest for each subject were our outcome maps for the group analysis. More specifically, the single-subject statistical maps with the contrast of interest were difference of beta coefficients from the regression of task minus control. Three group analyses were conducted: first, the main effect of task across all participants was tested in relation to WMH, HbA1c, and response time; this was done by taking a one-sample *t*-test across all subjects on the beta maps task-control which generates a *T*-statistic map. The relationship between HbA1c and WMH and activation (during condition) was also investigated by testing the null hypothesis that the slope WMH = 0 for our first model and slope median response time = 0 for our second model using a multiple linear regression model and testing the coefficient of interest using a *t*-test. The statistical maps representing the associations between fMRI activation and WMH used a threshold *T* = 3.19 (*p* < 0.001, uncorrected). To correct for multiple comparisons, permutation methods for peak-cluster level error correction were applied (AlphaSim). Specifically, we compute (via simulations) the probability of a random field of noise producing a cluster of a certain size given some threshold (*p* = 0.001). We found that a minimum cluster size of 130 voxels was needed to attain significance of *p* < 0.05, corrected.

## 3. Results

The characteristics of the participants included in this analysis (Table 1) are similar to those previously reported for this cohort [17, 25, 54–56].

### 3.1. Associations between Sample Characteristics and Response Time While Performing the DSST in the Scanner

Length of response time while performing the DSST in the scanner (e.g., longer time = worse performance) was directly associated with age, T1D duration, HbA1c months, burden of WMH, and higher prevalence of small vessel disease and inversely related to accuracy (Table 1). Of note, associations with HbA1c or glucose levels on the day of MRI were not significant (Table 1). Length of response time while performing the DSST in the scanner was also inversely associated with scores on cognitive tests performed outside the scanner, including the pencil and paper DSST and the composite score from the psychomotor speed domain (Table 1). Longer response time was also associated with higher probability of having clinically relevant cognitive impairment (Table 1). These associations remained significant after adjustment for age or duration (not shown).

### 3.2. Associations between Hyperglycemia and Response Time While Performing the DSST in the Scanner

In multivariable regression models (Table 2), higher HbA1c months remained associated with longer response time independent of adjustment for age, WMH, or other markers of small vessel diseases. Of note, the size of the regression coefficient was attenuated by >10% after adjustment for proliferative retinopathy while it remained similar after adjustment for other markers of small vessel disease (Table 2).

### 3.3. Main Effect

**Figure 1:**
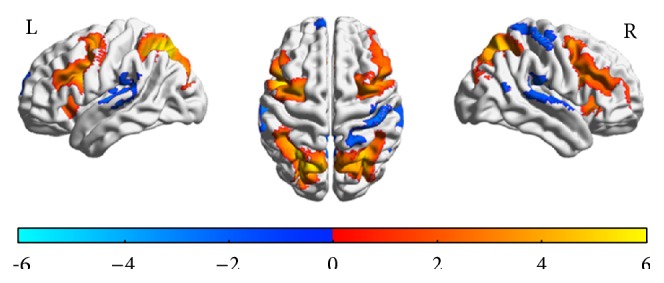
Spatial distribution of brain functional MRI activation during DSST performance in the scanner compared to performing the control condition. *T* maps of correlation analysis of task-related activity while performing the DSST versus control in the scanner for the left (L) and right (R) hemisphere. The color bar indicates the direction of association: red-yellow for positive correlations (e.g., higher activation for DSST-control contrast) and blue for negative correlations (e.g., higher activation for control-DSST contrast). Threshold was at *t* = 1.71; voxelwise alpha < 0.001; AlphaSim corrected at *p* < 0.05. See Table 3 for list of regions.

Brain activation while performing the DSST in the scanner was significantly greater than brain activation while performing the control condition in regions previously shown to be related to psychomotor speed in general and to this task in particular (Figure 1 and Table 3): dorsolateral prefrontal cortex which extended rostrally to include the inferior frontal gyrus, caudally to include pre- and postcentral gyri and the parietal cortex, and subcortically to include thalamus and dorsal cingulate gyrus. Analyses to test the opposite direction of association (main effect of control condition minus DSST) displayed a network of areas that extended from cortical temporal areas toward the posterior cingulate gyrus and medial frontal gyrus medially.

### 3.4. Associations between Hyperglycemia, Brain Activation While Performing the DSST in the Scanner, and Response Time

**Figure 2:**
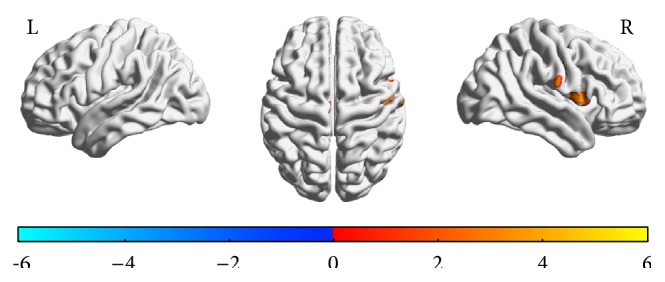
Spatial distribution of associations between hemoglobin A1c and brain functional MRI activation during DSST performance in the scanner. Activation was also present in the dorsal cingulate gyrus (not shown). *T* maps of correlation analysis of hemoglobin A1c with task-related activity while performing the DSST in the scanner for the left (L) and right (R) hemisphere. The color bar indicates the direction of association: red-yellow for positive correlations (e.g., higher activation for higher HbA1c) and blue for negative correlations (e.g., higher activation for lower HbA1c). Threshold was at *t* = 1.71; voxelwise alpha < 0.001; AlphaSim corrected at *p* < 0.05. See Table 2 for list of regions.

There was a significant and direct association between HbA1c months and brain activation while performing the task (Figure 2), which was localized in some of the regions related to the main effect: inferior frontal, precentral, and dorsal cingulate gyri in the right hemisphere (Table 4). Analyses were similar after adjustment for WMH and complications (not shown).

**Figure 3:**
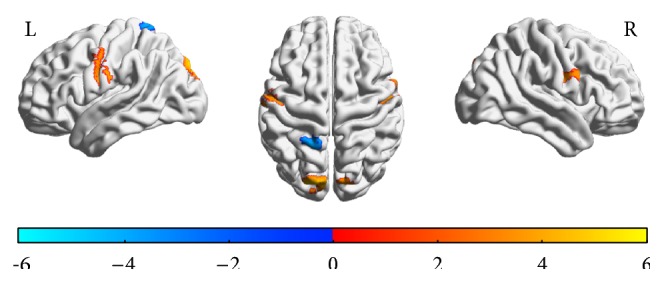
Spatial distribution of associations between response time and brain functional MRI activation during DSST performance in the scanner. Activation was also present in the thalamus (not shown). *T* maps of correlation analysis of response time with task-related activity while performing the DSST in the scanner for the left (L) and right (R) hemisphere. The color bar indicates the direction of association: red-yellow for positive correlations (e.g., higher activation for longer response time or slower performance) and blue for negative correlations (e.g., higher activation for shorter response time or faster performance). Threshold was at *t* = 1.71 and cluster probability (alpha) < 0.001. See Table 3 for list of regions.

There was a significant and direct association between brain activation and length of response time while performing the DSST (Figure 3), localized in the inferior frontal gyrus, precentral gyrus in the right hemisphere, the precentral gyrus in the left hemisphere, and the thalamus, postcentral gyrus, and cuneus bilaterally (Table 5). Additionally, there was a significant and inverse association between length of response time and brain activation while performing the DSST (Figure 3), localized in both the medial (precuneus) and lateral areas of the superior parietal lobule (Table 5).

### 3.5. Contribution of Brain Small Vessel Disease

Higher WMH burden was not associated with brain activation while performing the task in the scanner and the interaction of WMH burden and response time on brain activation while performing the task was also not significant (not shown).

## 4. Discussion

The results of this functional neuroimaging study suggest an explanatory pathway of slower psychomotor speed in middle-aged persons with childhood-onset T1D. We found that chronic hyperglycemia was related to higher activation in selected brain regions that were engaged in performing the DSST and to worse task performance. Moreover, we found a direct association between activation in some of these brain regions and performance. Contrary to our expectations, brain small vessel disease did not contribute to these relationships.

Understanding the mechanisms underlying slowing of psychomotor speed in persons with T1D is urgently needed. Psychomotor slowing reflects deficits in attention, planning, and problem-solving skills. It is a potent predictor of poorer diabetes management in adults with type 2 diabetes [6–8] and is associated with increased risk and fear of falls and greater psychological distress [6]. Psychomotor slowing is also a well-known risk factor for developing disability, dementia, and mortality in older persons without T1D [9, 10]; similar trends may be observed in future years for middle-aged persons with T1D, with rising personal, societal, and public health costs [57]. Both the increasing incidence rate [58] and longer life expectancy of persons with T1D [14] contribute to an increasing number of individuals with T1D who are aging. Therefore, addressing psychomotor slowing in these patients may help reduce the risk of developing these negative health outcomes.

The neurodegenerative effects of exposure to chronic hyperglycemia have been consistently shown. Chronic hyperglycemia appears to have a stronger impact on poor cognitive function for adults with T1D [17, 24] whereas hypoglycemia appears to play a more important role in the relationships with cognitive deficits in younger persons with T1D [59]. Consistent with the literature in adults with T1D, we found that chronic hyperglycemia was associated with poorer performance of a task of psychomotor speed while in the scanner, and this association was robust to adjustment for other covariates.

There appeared to be spatial colocalization of the regions that were more active in relationship to chronic hyperglycemia with the regions that were more active in the presence of slower performance, specifically the right inferior frontal and precentral gyri. One interpretation for these findings is that these regions are vulnerable to hyperglycemia and respond to this condition through hyperactivation, which in turn would negatively interfere with performance. Other studies have found an association between hyperglycemia and higher activation in these areas. A prior fMRI study [60] of visual spatial working memory showed that young persons with T1D (*n* = 16, mean age: 20 years) had higher brain activation compared to similarly aged persons without T1D (*n* = 16, age = 21 years) and this pattern of hyperactivation was localized in the right inferior frontal gyrus, cerebellum, basal ganglia, and substantia nigra. Another small study of newly diagnosed T2D [61] (mean age = 41, *n* = 12) showed hyperactivation while performing a working memory task in a network of prefrontoparietal regions that included the inferior frontal gyrus, as compared to controls (mean age: 40 years, *n* = 12). While the overlap of these studies' results with ours (i.e., hyperactivation in the inferior prefrontal gyrus) is of interest, direct comparisons cannot be done because these prior studies did not directly examine within-group relationships between chronic hyperglycemia, microvascular complications, brain activation, and performance.

Why would chronic hyperglycemia cause higher brain activation? Activation as measured in the scanner is the result of several interrelated physiological processes related to exposure to a mental challenge. Blood flow, neuronal activation, and ability of the neurons to extract oxygen all contribute to the intensity of the activation signal. A mechanism implicating small vessel disease has been suggested to explain the effect of chronic hyperglycemia on lower cognitive function and higher brain activation. Chronic hyperglycemia could disrupt the integrity of the blood brain barrier through several pathways [19], including basal membrane thickening of the small vessels, which would be manifested radiologically as WMH and could compromise efficient oxygen delivery and lead to changes in* brain activation* while performing a task in the scanner. Although we found a direct relationship between WMH and poorer performance, WMH did not appear to mediate the association between hyperglycemia and performance in the scanner nor did we find an association between WMH and brain activation. A positive relationship between WMH lesion load and higher brain activation in the frontal lobe has been found in some studies of older adults [28, 29]. Unfortunately, evidence in favor of (or against) these associations has been indirect for persons with T1D. We are aware of one prior functional neuroimaging study of 24 adults (mean age: 40 years) with T1D [20] showing that those with small vessel disease, measured as presence of proliferative retinopathy, had higher task-related activation in the orbital frontal gyrus. It is possible that, in our cohort, WMH is not severe enough to interfere with the task we used in the scanner and/or that this task is too easy. Indeed, most participants performed the task with high accuracy. Another possibility is that the spatial distribution of WMH is not affecting the areas critical for performing this task. It would be informative to examine the patterns of activation in relationship with WMH while performing other tasks of psychomotor speed that are titrated for difficulty, as well as to examine the spatial distribution of WMH.

One other potential pathway linking hyperglycemia to higher activation could be via greater production of the excitatory neurotransmitter glutamate. It is known that hyperglycemia is related to higher levels of glutamate and a relationship between hyperglycemia, higher glutamate, and higher brain activation in the frontal lobe has been shown in patients with T1D [62]. Chronic exposure to glutamate is known to cause neurotoxicity; hence, higher activation related to higher glutamate could also explain the relationship between higher activation and lower performance.

The association between hyperglycemia and higher activation could also be due to the direct toxic effects of higher levels of glucose at time of testing. Prior studies examining the effect of transient, acute changes in glucose levels on brain activation, both within and outside physiological ranges, have mostly focused on hypoglycemia and have yielded conflicting results. Some studies have found hypoglycemia is related to lower brain activation [63, 64] while others have found that acute, transient hypoglycemia is related to higher activation [65, 66]. We did not find an association between glucose or HbA1c levels on day of testing with brain activation or performance; thus, it is more likely that the effects observed in our study would be due to longer-term exposure to hyperglycemia.

It remains unclear whether brain activation and performance are monotonically related [67, 68]. Higher activation has been found in the presence of higher performance, possibly to compensate for other deficits, and also in relationship with lower performance in persons with Alzheimer's disease, as a sign of dedifferentiation. In general, higher activation in the presence of lower performance has been postulated to represent signs of dysregulation of neuronal activation. In our study, we found a dual pattern of activation in relationship to performance, consisting of inverse associations in cortico-subcortical networks that included those regions related to chronic hyperglycemia (e.g., inferior frontal gyrus, precentral gyrus), as well as direct associations with activation in the left superior parietal lobule. The direct association between performance and superior parietal activation is consistent with what we have previously found in a group of older adults with a range of chronic health conditions [38, 39]. In this prior study of older adults without T1D, we also found that activation in the superior parietal cortex was higher for those adults who maintained higher performance in the presence of higher WMH burden. Thus, higher activation in the superior parietal lobe may be compensating for abnormalities occurring in other tissues (e.g., presence of WMH in the study of older adults) and/or for the dysregulation of activation in regions vulnerable to hyperglycemia (e.g., hyperactivation of inferior frontal gyrus and precentral gyrus in the presence of hyperglycemia in this study). If confirmed in other studies, it would be very informative to examine the factors most related to integrity of the superior parietal lobe and whether improvement of activation in the superior parietal lobe would also improve psychomotor speed.

Strengths of our study include the large sample size and the extensive characterization of cognitive function using pencil and paper tasks, as well as information on complications obtained over prior years. The performance measure that we chose in this study, length of response time in the scanner, appeared to effectively capture the severity of impairment in psychomotor speed and the presence of cognitive impairment as shown by the strong correlations with the score on the pencil and paper tests. Another strength of this study is that accuracy and reaction time of the participants were measured while recording their actual brain signal, thus allowing correlation of performance with brain activation. Results of this study should be considered with caution, especially with regard to generalization of results. It is likely that this group of patients, who have lived most of their lives with T1D into their fifth and sixth decade of life, may harbor unique characteristics compared to younger persons with T1D. Moreover, we did not test all the potential pathways linking hyperglycemia with brain activation. A limitation inherent in all task-related fMRI studies is that results are task specific. It is possible that a different task of psychomotor speed would reveal a different spatial distribution of hyperactivation.

## 5. Conclusions

Additional studies are needed to assess whether patterns of brain activation would be helpful markers of cognitive complications in persons with T1D. It would also be important to know whether better glycemic control improves cognitive function via changes in the patterns of brain activation. This would not only have tremendous implications to further our understanding of the mechanisms underlying psychomotor speed but can also help understand whether the brains of persons with T1D retain plasticity and reserve.

## Figures and Tables

**Table 1:** Characteristics of participants (*N* = 83) and associations with median response time while performing the task (DSST) in the scanner (Spearman or Pearson). Measures are from day of MRI (2010–2013) unless otherwise specified.

		Mean ± SD or *N* (%)	Correlation coefficient, *p* values
Demographic characteristics	Age at MRI (years)	49.04 ± 6.89	−0.37, <0.001
	Education (years)	15 ± 2	−0.09, 0.38
	Female	42 (48%)	0.12, 0.27

Diabetes-related factors and complications	T1D duration at MRI (years)	40.80 ± 6.26	0.28, 0.01
	Age at T1D diagnosis (years)	8.24 ± 4.25	0.18, 0.10
	Serum glucose (mg/dL)	177.52 ± 90.68	−0.06, 0.61
	HbA1c (%)	7.96 ± 2.32	0.15, 0.17
	HbA1c months (AU)	971.15 ± 374.24	0.32, <0.001
	Confirmed distal symmetric polyneuropathy^*∗*^	38 (48%)	0.38, <0.001
	Overt nephropathy^*∗*^	23 (32%)	0.33, <0.001
	Proliferative retinopathy^*∗*^	39 (45%)	0.36, <0.001

MRI measures	Correct median response time (ms)	1353.39 ± 322.12	—
	White matter hyperintensity volume (% total brain volume)	0.002 ± 0.003	0.24, 0.02
	White matter hyperintensity severity, Fazekas rating = 3	10 (11%)	0.25, 0.02

Cognitive measures	NAART (verbal IQ estimate)	108 ± 7	−0.17, 0.14
	Digit symbol substitution test, number complete in 90 seconds (pencil and paper test)	56 ± 13	−0.56, <0.001
	Information processing domain, *z*-score	0.54 ± 0.82	−0.57, <0.001
	Clinically relevant cognitive impairment	28 (35%)	0.37, <0.001

^*∗*^Documented in 2004–2006.

**Table 2:** Age-adjusted associations between HbA1c months and DSST response time while in the scanner.

	Age-adjusted betas (standard errors), *p* value
Model 1	0.249 (0.075), *p* = 0.001
Model 2, adjusted for WMH (Fazekas)	0.261 (0.074), *p* = 0.001
Model 3, adjusted for distal symmetric polyneuropathy^∧^	0.221 (0.075), *p* = 0.005
Model 4, adjusted for overt nephropathy^∧^	0.265 (0.088), *p* = 0.004
Model 5, adjusted for proliferative retinopathy^∧^	0.171 (0.078), *p* = 0.032

^∧^Adjusted for interval of time between date of documentation of that complication (2004–2006) and date of MRI (2010–2013).

**Table 3:** Regions with functional activation that were correlated with performing the task in the scanner (main effect): positive associations are listed first and negative associations are listed next.

Regions	Cluster size	Peak *T*-Score (df = 84)	Montreal Neurological Institute coordinate for Peak *T*-Score
Regions with activation positively correlated with response time			
Superior parietal lobe, bilaterally	6912	12	−28, −62, 52^1^
Left middle frontal gyrus	3529	12	−44, 2, 34^2^
Right middle frontal gyrus	3133	8.5	36, 2, 52^3^
Medial frontal gyrus, bilaterally	1196	8.6	2, 20, 46^4^
Right inferior frontal gyrus	375	6.3	34, 24, −5^5^
Right thalamus	214	4	18, −6, 19
Left thalamus	212	4.1	−16, −12, 16

Regions with activation negatively correlated with response time			
Left superior temporal gyrus	1285	−6.6	−66, −22, 1
Right superior temporal gyrus	891	−5	56, −30, 22
Posterior cingulate cortex, bilaterally	821	−6.1	−4, −52, 28
Left medial frontal gyrus	420	−5.1	−2, 64, 1
Right postcentral gyrus	418	−5.9	48, −26, 64

This table reports the spatial distribution of the mean group activation (obtained from the DSST > control condition contrast and from the control condition > DSST contrast), including the size of cluster, the maximum *Z* statistic for the cluster, and the location of the maximum *Z* statistic in Montreal Neurological Institute coordinates. The corrected alpha is the probability of false positive detection based on the combination of individual voxel probability thresholding and minimum cluster size thresholding.

^1^This cluster extends medially to include the precuneus and caudally to include the superior occipital gyrus; it includes the most dorsal part of the inferior parietal lobule.

^2^This cluster extends rostrally to include the supplementary motor area, caudally to include the precentral gyrus, and medially to include the insula.

^3^This cluster extends rostrally to include the supplementary motor area, caudally to include the precentral gyrus, and ventrally to include the inferior frontal gyrus.

^4^This cluster extends caudally in the right hemisphere to include the dorsal cingulate cortex.

^5^This cluster covers part of the insula.

**Table 4:** Regions with functional activation that were positively correlated with hemoglobin A1c months. No significant voxels were negatively correlated with hemoglobin A1c months.

Regions	Cluster size	Peak *T*-Score (df = 84)	Montreal Neurological Institute coordinate for Peak *T*-Score
Right dorsal cingulate gyrus	274	3.58	26, 2, 34
Right precentral gyrus	267	3.65	50, −8, 31^1^
Right inferior frontal gyrus, pars orbitalis	134	3.55	50, 14, 7

This table reports the spatial distribution of the association of hemoglobin A1c months with the mean group activation (obtained from the DSST > control condition contrast), including the size of cluster, the maximum *Z* statistic for the cluster, and the location of the maximum *Z* statistic in Montreal Neurological Institute coordinates. The corrected alpha is the probability of false positive detection based on the combination of individual voxel probability thresholding and minimum cluster size thresholding.

^1^This cluster extended caudally to the right postcentral gyrus.

**Table 5:** Regions where functional activation correlated with response time.

Regions	Cluster size	Peak *T*-Score (df = 81)	Montreal Neurological Institute coordinate for Peak *T*-Score
Regions with activation positively correlated with response time			
Right inferior frontal gyrus	958	4.40	2, −12, 4^1^
Left precentral gyrus	654	4.57	−62, −10, 28^2^
Left superior occipital gyrus	366	6.05	−18, −92, 34^3^

Regions with activation negatively correlated with response time			
Left superior parietal lobule	130	−3.95	−20, −50, 73^4^

This table reports the spatial distribution of the association of length of response time with the mean brain activation (obtained from the DSST > control condition contrast), including the size of cluster, the maximum *Z* statistic for the cluster, and the location of the maximum *Z* statistic in Montreal Neurological Institute coordinates. The corrected alpha is the probability of false positive detection based on the combination of individual voxel probability thresholding and minimum cluster size thresholding.

^1^This cluster includes the lower portion of the right pre- and postcentral gyri and it extended medially to include the thalamus.

^2^This cluster extended caudally to include the left postcentral gyrus.

^3^This cluster extends rostrally to include the left and right cuneus.

^4^This cluster includes the left precuneus.

## References

[1] Ryan C. M., Geckle M. O., Orchard T. J. (2003). Cognitive efficiency declines over time in adults with Type 1 diabetes: effects of micro- and macrovascular complications. *Diabetologia*.

[2] McCarthy A. M., Lindgren S., Mengeling M. A., Tsalikian E., Engvall J. C. (2002). Effects of diabetes on learning in children. *Pediatrics*.

[3] Ferguson S. C., Blane A., Wardlaw J. (2005). Influence of an early-onset age of type 1 diabetes on cerebral structure and cognitive function. *Diabetes Care*.

[4] Brands A. M. A., Kessels R. P. C., Hoogma R. P. L. M. (2006). Cognitive performance, psychological well-being, and brain magnetic resonance imaging in older patients with type 1 diabetes. *Diabetes*.

[5] Biessels G.-J. (1999). Cerebral complications of diabetes: clinical findings and pathogenetic mechanisms. *The Netherlands Journal of Medicine*.

[6] Munshi M. N., Hayes M., Iwata I., Lee Y., Weinger K. (2012). Which aspects of executive dysfunction influence ability to manage diabetes in older adults?. *Diabetic Medicine*.

[7] Primožič S., Tavčar R., Avbelj M., Dernovšek M. Z., Oblak M. R. (2012). Specific cognitive abilities are associated with diabetes self-management behavior among patients with type 2 diabetes. *Diabetes Research and Clinical Practice*.

[8] Feil D. G., Zhu C. W., Sultzer D. L. (2012). The relationship between cognitive impairment and diabetes self-management in a population-based community sample of older adults with type 2 diabetes. *Journal of Behavioral Medicine*.

[9] Rosano C., Chang Y.-F., Kuller L. H. (2013). Long-term survival in adults 65 years and older with white matter hyperintensity: association with performance on the digit symbol substitution test. *Psychosomatic Medicine*.

[10] Rosano C., Newman A. B., Katz R., Hirsch C. H., Kuller L. H. (2008). Association between lower digit symbol substitution test score and slower gait and greater risk of mortality and of developing incident disability in well-functioning older adults. *Journal of the American Geriatrics Society*.

[11] Tonoli C., Heyman E., Roelands B. (2005). Type 1 diabetes-associated cognitive decline: a meta-analysis and update of the current literature. *Journal of Diabetes*.

[12] Brands A. M. A., Biessels G.-J., De Haan E. H. F., Kappelle L. J., Kessels R. P. C. (2005). The effects of type 1 diabetes on cognitive performance: a meta-analysis. *Diabetes Care*.

[13] McCrimmon R. J., Ryan C. M., Frier B. M. (2012). Diabetes and cognitive dysfunction. *The Lancet*.

[14] Miller R. G., Secrest A. M., Sharma R. K., Songer T. J., Orchard T. J. (2012). Improvements in the life expectancy of type 1 diabetes: the pittsburgh epidemiology of diabetes complications study cohort. *Diabetes*.

[15] Cameron N. E., Eaton S. E. M., Cotter M. A., Tesfaye S. (2001). Vascular factors and metabolic interactions in the pathogenesis of diabetic neuropathy. *Diabetologia*.

[16] Sheetz M. J., King G. L. (2002). Molecular understanding of hyperglycemia's adverse effects for diabetic complications. *The Journal of the American Medical Association*.

[17] Nunley K. A., Rosano C., Ryan C. M. (2015). Clinically relevant cognitive impairment in middle-aged adults with childhood-onset type 1 diabetes. *Diabetes Care*.

[18] Wessels A. M., Scheltens P., Barkhof F., Heine R. J. (2008). Hyperglycaemia as a determinant of cognitive decline in patients with type 1 diabetes. *European Journal of Pharmacology*.

[19] Brownlee M. (2001). Biochemistry and molecular cell biology of diabetic complications. *Nature*.

[20] Wessels A. M., Rombouts S. A. R. B., Simsek S. (2006). Microvascular disease in type 1 diabetes alters brain activation: a functional magnetic resonance imaging study. *Diabetes*.

[21] Brismar T., Maurex L., Cooray G. (2007). Predictors of cognitive impairment in type 1 diabetes. *Psychoneuroendocrinology*.

[22] van Duinkerken E., Schoonheim M. M., Sanz-Arigita E. J. (2012). Resting-state brain networks in type 1 diabetic patients with and without microangiopathy and their relation to cognitive functions and disease variables. *Diabetes*.

[23] Ferguson S. C., Blane A., Perros P. (2003). Cognitive ability and brain structure in type 1 diabetes: relation to microangiopathy and preceding severe hypoglycemia. *Diabetes*.

[24] Jacobson A. M., Ryan C. M., Cleary P. A. (2011). Biomedical risk factors for decreased cognitive functioning in type 1 diabetes: an 18 year follow-up of the Diabetes Control and Complications Trial (DCCT) cohort. *Diabetologia*.

[25] Nunley K. A., Ryan C. M., Orchard T. J. (2015). White matter hyperintensities in middle-aged adults with childhood-onset type 1 diabetes. *Neurology*.

[26] Johnson P., Brendel K., Meezan E. (1982). Thickened cerebral cortical capillary basement membranes in diabetics. *Archives of Pathology & Laboratory Medicine*.

[27] McKiernan K. A., Kaufman J. N., Kucera-Thompson J., Binder J. R. (2003). A parametric manipulation of factors affecting task-induced deactivation in functional neuroimaging. *Journal of Cognitive Neuroscience*.

[28] Aizenstein H. J., Andreescu C., Edelman K. L. (2011). fMRI correlates of white matter hyperintensities in late-life depression. *The American Journal of Psychiatry*.

[29] Lockhart S. N., Luck S. J., Geng J. (2015). White matter hyperintensities among older adults are associated with futile increase in frontal activation and functional connectivity during spatial search. *PLoS ONE*.

[30] Patel M. J., Boada F. E., Price J. C. (2012). Association of small vessel ischemic white matter changes with BOLD fMRI imaging in the elderly. *Psychiatry Research—Neuroimaging*.

[31] Pambianco G., Costacou T., Ellis D., Becker D. J., Klein R., Orchard T. J. (2006). The 30-year natural history of type 1 diabetes complications: the Pittsburgh epidemiology of diabetes complications study experience. *Diabetes*.

[32] Orchard T. J., Forrest K. Y.-Z., Ellis D., Becker D. J. (1997). Cumulative glycemic exposure and microvascular complications in insulin-dependent diabetes mellitus: the glycemic threshold revisited. *Archives of Internal Medicine*.

[33] Koenig R. J., Peterson C. M., Jones R. L., Saudek C., Lehrman M., Cerami A. (1976). Correlation of glucose regulation and hemoglobin A_Ic_ in diabetes mellitus. *The New England Journal of Medicine*.

[34] Venkatraman V. K., Aizenstein H. J., Newman A. B. (2011). Lower digit symbol substitution score in the oldest old is related to magnetization transfer and diffusion tensor imaging of the white matter. *Frontiers in Aging Neuroscience*.

[35] Wu M., Rosano C., Butters M. (2006). A fully automated method for quantifying and localizing white matter hyperintensities on MR images. *Psychiatry Research*.

[36] Salthouse T. A. (1978). The role of memory in the age decline in digit-symbol substitution performance. *Journals of Gerontology*.

[37] Matarazzo J. D., Herman D. O. (1984). Base rate data for the WAIS-R: test-retest stability and VIQ-PIQ differences.. *Journal of Clinical Neuropsychology*.

[38] Rosano C., Venkatraman V. K., Guralnik J. (2010). Psychomotor speed and functional brain MRI 2 years after completing a physical activity treatment. *Journals of Gerontology Series A—Biological Sciences and Medical Sciences*.

[39] Venkatraman V. K., Aizenstein H., Guralnik J. (2010). Executive control function, brain activation and white matter hyperintensities in older adults. *NeuroImage*.

[40] Lezak M. D. (2004). *Neuropsychological Assessment*.

[41] Anastasi A. (1958). *Differential Psychology*.

[42] Stephens R., Sreenivasan B. (2002). Analysis of substitution test performance using eye movement and video data. *Applied Neuropsychology*.

[43] Gilmore G. C., Royer F. L., Gruhn J. J., Esson M. J. (2004). Symbol-digit substitution and individual differences in visual search ability. *Intelligence*.

[44] Wager T. D., Smith E. E. (2003). Neuroimaging studies of working memory: a meta-analysis. *Cognitive, Affective & Behavioral Neuroscience*.

[45] Baddeley A. (2003). Working memory: looking back and looking forward. *Nature Reviews Neuroscience*.

[46] LaBar K. S., Gitelman D. R., Parrish T. B., Mesulam M.-M. (1999). Neuroanatomic overlap of working memory and spatial attention networks: a functional MRI comparison within subjects. *NeuroImage*.

[47] Smith E. E., Jonides J. (1997). Working memory: a view from neuroimaging. *Cognitive Psychology*.

[48] Udupa J. K., Samarasekera S. (1996). Fuzzy connectedness and object definition: theory, algorithms, and applications in image segmentation. *Graphical Models and Image Processing*.

[49] Strauss E., Sherman E. M. S., Spreen O. (2006). *A Compendium of Neuropsychological Tests: Administrations, Norms and Commentary*.

[50] Ardila A. (2007). Normal aging increases cognitive heterogeneity: analysis of dispersion in WAIS-III scores across age. *Archives of Clinical Neuropsychology*.

[51] Dore G. A., Elias M. F., Robbins M. A., Elias P. K., Brennan S. L. (2007). Cognitive performance and age: norms from the Maine-Syracuse study. *Experimental Aging Research*.

[52] MacKinnon D. P., Fairchild A. J., Fritz M. S. (2007). Mediation analysis. *Annual Review of Psychology*.

[53] Friston K. J., Holmes A. P., Worsley K. J., Poline J.-P., Frith C. D., Frackowiak R. S. J. (1994). Statistical parametric maps in functional imaging: a general linear approach. *Human Brain Mapping*.

[54] Costacou T., Rosano C., Aizenstein H. (2015). The haptoglobin 1 allele correlates with white matter hyperintensities in middle-aged adults with type 1 diabetes. *Diabetes*.

[55] Hughes T. M., Ryan C. M., Aizenstein H. J. (2013). Frontal gray matter atrophy in middle aged adults with type 1 diabetes is independent of cardiovascular risk factors and diabetes complications. *Journal of Diabetes and Its Complications*.

[56] Ryan J. P., Aizenstein H. J., Orchard T. J. (2015). Age of childhood onset in type 1 diabetes and functional brain connectivity in midlife. *Psychosomatic Medicine*.

[57] Ozieh M. N., Bishu K. G., Dismuke C. E., Egede L. E. (2015). Trends in health care expenditure in U.S. adults with diabetes: 2002–2011. *Diabetes Care*.

[58] Menke A., Orchard T. J., Imperatore G., Bullard K. M., Mayer-Davis E., Cowie C. C. (2013). The prevalence of type 1 diabetes in the United States. *Epidemiology*.

[59] Northam E. A., Lin A. (2010). Hypoglycaemia in childhood onset type 1 diabetes—part villain, but not the only one. *Pediatric Diabetes*.

[60] Gallardo-Moreno G. B., González-Garrido A. A., Gudayol-Ferré E., Guàrdia-Olmos J. (2015). Type 1 diabetes modifies brain activation in young patients while performing visuospatial working memory tasks. *Journal of Diabetes Research*.

[61] He X.-S., Wang Z.-X., Zhu Y.-Z. (2015). Hyperactivation of working memory-related brain circuits in newly diagnosed middle-aged type 2 diabetics. *Acta Diabetologica*.

[62] Lyoo I. K., Yoon S. J., Musen G. (2009). Altered prefrontal glutamate-glutamine-gamma-aminobutyric acid levels and relation to low cognitive performance and depressive symptoms in type 1 diabetes mellitus. *Archives of General Psychiatry*.

[63] Driesen N. R., Goldberg P. A., Anderson A. W. (2007). Hypoglycemia reduces the blood-oxygenation level dependent signal in primary auditory and visual cortex: a functional magnetic resonance imaging study. *Journal of Neuroscience Research*.

[64] Rosenthal J. M., Amiel S. A., Yágüez L. (2001). The effect of acute hypoglycemia on brain function and activation: a functional magnetic resonance imaging study. *Diabetes*.

[65] Musen G., Simonson D. C., Bolo N. R. (2008). Regional brain activation during hypoglycemia in type 1 diabetes. *The Journal of Clinical Endocrinology & Metabolism*.

[66] Bolo N. R., Musen G., Jacobson A. M. (2011). Brain activation during working memory is altered in patients with type 1 diabetes during hypoglycemia. *Diabetes*.

[67] Cabeza R., Anderson N. D., Locantore J. K., McIntosh A. R. (2002). Aging gracefully: compensatory brain activity in high-performing older adults. *NeuroImage*.

[68] Kennedy K. M., Rodrigue K. M., Bischof G. N., Hebrank A. C., Reuter-Lorenz P. A., Park D. C. (2015). Age trajectories of functional activation under conditions of low and high processing demands: an adult lifespan fMRI study of the aging brain. *NeuroImage*.

