# Corrigendum to “Brain Activation and Psychomotor Speed in Middle-Aged Patients with Type 1 Diabetes: Relationships with Hyperglycemia and Brain Small Vessel Disease”

**DOI:** 10.1155/2017/4295645

**Published:** 2017-03-16

**Authors:** Misun Hwang, Dana L. Tudorascu, Karen Nunley, Helmet Karim, Howard J. Aizenstein, Trevor J. Orchard, Caterina Rosano

**Affiliations:** ^1^Department of Radiology, University of Pittsburgh, 3600 Forbes Avenue, Plaza Level, Pittsburgh, PA 15213, USA; ^2^Department of Internal Medicine, Department of Psychiatry, and Department of Biostatistics, University of Pittsburgh, 200 Meyran Avenue, Suite 326, Pittsburgh, PA 15213, USA; ^3^Department of Epidemiology, University of Pittsburgh, 130 N. Bellefield Avenue, Suite 443, Pittsburgh, PA 15213, USA; ^4^Department of Bioengineering, University of Pittsburgh, 253 Sterling Plaza, Pittsburgh, PA 15213, USA; ^5^Department of Psychiatry, University of Pittsburgh, 3811 O'Hara Street, Pittsburgh, PA 15213, USA; ^6^Department of Epidemiology, University of Pittsburgh, 3512 Fifth Avenue, Pittsburgh, PA 15213, USA; ^7^Department of Epidemiology, University of Pittsburgh, 130 N. Bellefield Avenue, Suite 467, Pittsburgh, PA 15213, USA

 In the article titled “Brain Activation and Psychomotor Speed in Middle-Aged Patients with Type 1 Diabetes: Relationships with Hyperglycemia and Brain Small Vessel Disease” [1], there was an error in the subheadings of Table 3. The phrase “response time” should be replaced with the phrase “main effect.” The correct table is shown below.

## Figures and Tables

**Table 3:** Regions with functional activation that were correlated with performing the task in the scanner (main effect): positive associations are listed first and negative associations are listed next.

Regions	Cluster size	Peak *T*-Score (df = 84)	Montreal Neurological Institute coordinate for Peak *T*-Score
Regions with activation positively correlated with main effect			
Superior parietal lobe, bilaterally	6912	12	−28, −62, 52^1^
Left middle frontal gyrus	3529	12	−44, 2, 34^2^
Right middle frontal gyrus	3133	8.5	36, 2, 52^3^
Medial frontal gyrus, bilaterally	1196	8.6	2, 20, 46^4^
Right inferior frontal gyrus	375	6.3	34, 24, −5^5^
Right thalamus	214	4	18, −6, 19
Left thalamus	212	4.1	−16, −12, 16

Regions with activation negatively correlated with main effect			
Left superior temporal gyrus	1285	−6.6	−66, −22, 1
Right superior temporal gyrus	891	−5	56, −30, 22
Posterior cingulate cortex, bilaterally	821	−6.1	−4, −52, 28
Left medial frontal gyrus	420	−5.1	−2, 64, 1
Right postcentral gyrus	418	−5.9	48, −26, 64

This table reports the spatial distribution of the mean group activation (obtained from the DSST > control condition contrast and from the control condition > DSST contrast), including the size of cluster, the maximum *Z* statistic for the cluster, and the location of the maximum *Z* statistic in Montreal Neurological Institute coordinates. The corrected alpha is the probability of false positive detection based on the combination of individual voxel probability thresholding and minimum cluster size thresholding.

^1^This cluster extends medially to include the precuneus and caudally to include the superior occipital gyrus; it includes the most dorsal part of the inferior parietal lobule.

^2^This cluster extends rostrally to include the supplementary motor area, caudally to include the precentral gyrus, and medially to include the insula.

^3^This cluster extends rostrally to include the supplementary motor area, caudally to include the precentral gyrus, and ventrally to include the inferior frontal gyrus.

^4^This cluster extends caudally in the right hemisphere to include the dorsal cingulate cortex.

^5^This cluster covers part of the insula.

## References

[1] Hwang M., Tudorascu D. L., Nunley K. (2016). Brain activation and psychomotor speed in middle-aged patients with type 1 diabetes: relationships with hyperglycemia and brain small vessel disease. *Journal of Diabetes Research*.

